# Barriers and Facilitators in the Uptake of Integrated Care Pathways for Older Patients by Healthcare Professionals: A Qualitative Analysis of the French National “Health Pathway of Seniors for Preserved Autonomy” Pilot Program: Barriers and Facilitators for Adhesion of Healthcare Professionals in Integrated Care for Older Patients: A Qualitative Assessment Based on the French National Experiment: ‘Health Pathway of Seniors for Preserved Autonomy’ (PAERPA)

**DOI:** 10.5334/ijic.5483

**Published:** 2021-04-22

**Authors:** Lorette Averlant, Mathieu Calafiore, François Puisieux, Claire Ramez, Fanny Sarrazin, Maxime Lotin, Romain Naessens, Apolline Delesalle, Gracia Adotey, Pascal Harduin, Nathalie Leveque, Delphine Dambre, Marguerite-Marie Defebvre, Carla Di Martino, Jean-Baptiste Beuscart

**Affiliations:** 1 Univ. Lille, CHU Lille, METRICS – ULR 2694: Evaluation des technologies de santé et des pratiques médicales, F-59000 Lille, France; 2 Department of General Medicine, Lille School of Medicine, Lille, France; 3 Service médecine polyvalente, Centre Hospitalier de Saint-Amand-les-Eaux, France; 4 Agence Régionale de Santé (ARS) Hauts-de-France, Lille, France

**Keywords:** integrated care, care pathway, elderly, frailty

## Abstract

**Introduction:**

Integrated care is a particularly promising approach in geriatrics – a field in which the medical, psychological and social issues are often complex. The uptake of integrated care by healthcare professionals (HCPs) is essential but varies markedly. The objective of the present study of healthcare professionals was to identify barriers to and facilitators of commitment to integrated care for seniors.

**Methods:**

We performed a two-step, qualitative study, comprising (i) six qualitative, semi-directive series of interviews with HCPs (hospital practitioners, family physicians, nurses and pharmacists) who agreed or disagreed to take part in the French national “Health Pathway of Seniors for Preserved Autonomy” (PAERPA) pilot program; and (ii) an analysis of the pooled results, in order to identify common concerns among the healthcare professionals.

**Results:**

We identified four key “barrier” and “facilitator” topics shared by HCPs who had committed to the pilot program and those who had not: (i) awareness of and/or interest in geriatric medicine and team working, (ii) the presence of a care coordinator; (iii) the provision of information about the program and about the patient, and communication between HCPs, and (iv) personal benefits for the HCPs and the patients.

**Key conclusions:**

The four key topics identified in this large qualitative study of several healthcare professions should be considered during the design and dissemination of integrated care pathways for older patients.

## Introduction

Healthcare for frail older adults is often compartmentalized by the care providers, and can thus become an uncoordinated succession of assessments and actions [1,2,3,4,5]. The objective of integrated care pathways is to improve patient care by coordinating existing devices and services. This approach appears to be relevant for the management of frail older patients.

The level of commitment by healthcare professionals (HCPs) is a key success factor for integrated care pathways. Unfortunately, the HCPs’ commitment to and participation in integrated care pathways are variable [6,7,8]. Several reviews of the scientific literature have synthesized and structured barriers and facilitators for the implementation of integrated care, including for older patients [4,9,10]. Most of these reviews have structured their findings at three levels: macro (system level), meso (professional, functional and organisational integration), and micro (clinical or service integration). This classification is notably based on conceptual frameworks, such as the one proposed by Valentjin et al [11]. Studies about barriers and facilitators for the involvement of HCPs in integrated care for frail older patients (micro and meso levels) were based on small numbers of HCPs or pilot projects, and mainly focused on family physicians [12,13,14,15].

The French national “Health Pathway of Seniors for Preserved Autonomy” (PAERPA) pilot program was initiated in 2014. It promotes the development of an integrated care pathway for the management of such frail patients aged 75 or over. The involvement of at least two different HCPs (including family physicians, hospital physicians, community pharmacists, and nurses) was required. The definition of integrated care proposed by Kodner was: “*Integration is a coherent set of methods and models on the funding, administrative, organisational, service delivery and clinical levels designed to create connectivity, alignment and collaboration within and between the cure and care sectors. The goal of these methods and models is to enhance quality of care and quality of life, consumer satisfaction and system efficiency for patients with complex, long term problems cutting across multiple services, providers and settings. The result of such multi-pronged efforts to promote integration for the benefit of these special patient groups is called ‘integrated care*” [16]. In relation to this definition, the integrated care pathway in PAERPA focused specifically on the organization and care practices for the coordination of health professionals in the care of the elderly, corresponding to “service delivery and clinical level and connectivity and collaboration within and between the cure and care sectors” [16].

The objective of the present study work was to identify the barriers to and facilitators for the involvement of HCPs in the implementation and maintenance of an integrated care pathway for frail older people (micro and meso levels).

## Material and Method

### Study design

**Figure 1:**
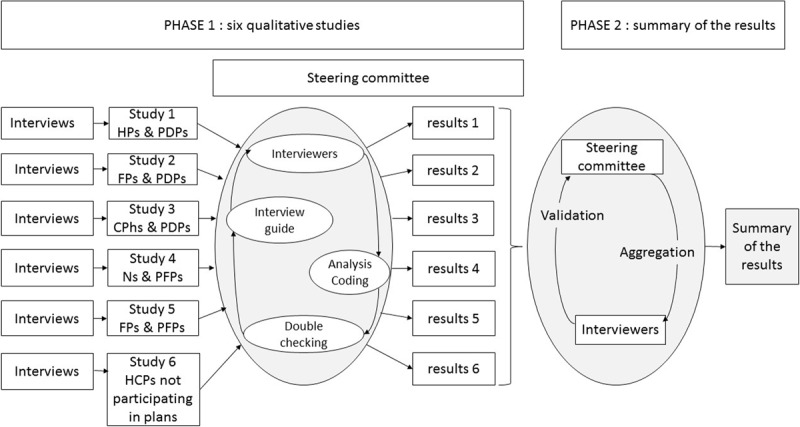
A schematic diagram of the two-phase study. In the first stage, six qualitative studies were carried out with four different HCP categories; in the second stage, the data were summarized. HP = hospital physician; FP = family physician; CPh = community pharmacist; N = Nurse; PDP = personalized drug plan; PFP = personalized frailty plan.

We performed a prospective, qualitative study of HCPs who agreed or refused to be involved in an integrated care pathway for frail older people. The study was conducted in two phases between December 2016 and September 2018. The methodological process is summarized in ***Figure 1***.

In the first phase of the study, seven investigators (ML, CR, FS, RN, PH, GA, and AD) carried out qualitative studies of four different categories of HCPs. All the investigators were medical interns or pharmacy students who had attended a standardized two-day training course on qualitative research at the Lille Faculty of Medicine (Lille, France). None of the investigators had a prior relationship with the interviewees. The investigators introduced themselves by explaining that the survey was being conducted as part of their thesis project.

The qualitative studies were conducted in parallel and were coordinated by a steering committee (JBB, MC, LA, and CDM). All of these qualitative studies were part of the main study and can be considered sub-studies. In monthly meetings with the seven investigators throughout the study period, the committee resolved any questions or discrepancies by consensus.

In the second phase of the study, the results of the qualitative studies were synthesized by the steering committee. The results of the six qualitative studies were aggregated, theorized, validated with each of the investigators, and checked against the verbatim.

The work was reported in accordance with the Consolidated Criteria for Reporting Qualitative Research (COREQ). Thirty of the 32 items on the COREQ checklist were completed (see Supplementary Data 1).

### Ethics

The HCPs gave their written, informed consent to participation in the study. Audio recordings were destroyed after transcription. As this type of study is not subject to the French legislation on clinical trials (government decree 2016–1537, dated November 16^th^ 2016), neither registration with the national data protection commission nor approval by an institutional review board was necessary [17].

### The French national “Health Pathway of Seniors for Preserved Autonomy” (PAERPA) pilot program

Between October 2014 and December 2019, the PAERPA pilot program was implemented in 16 regions of France by the Ministry of Social Affairs and Health. The program’s objectives were to (i) increase the relevance and quality of the care and support given to frail older patients (aged 75 and over), (ii) improve quality of life for older patients and their caregivers, and (iii) increase the overall efficiency of the care process. To this end, PAERPA promoted the development of an integrated care pathway by coordinating and meta-managing the operational stakeholders from various disciplines and various sectors (i.e. the health, medical and social sectors). Actions were implemented in order to modify current practices, train the stakeholders, and create (if required) new healthcare professions. In particular, a support center was set up to inform HCPs and users about this new opportunity in medical and social care, and to help them set up the requisite systems and carry out the various actions. Care coordinators were specifically recruited to help the HCPs and coordinate the PAERPA activities on the administrative level.

One of PAERPA’s major initiatives was the development of a personalized health pathway coordinated by a family physician and involving at least one other HCP (usually a nurse or a community pharmacist). Together, these professionals developed an integrated, personalized, multi-HCP health plan for each patient. The plan was signed by the HCPs and by the patient.

There were two types of personalized health plan:

– A personal drug plan (PDP) focused on the risk of adverse drug events. The PDPs were initiated during a hospital stay, continued in the community setting, and always included a community pharmacist.
– A personal frailty plan (PFP) focused on the prevention of frailty. The PFP were developed and implemented solely in a community setting, and involved a family physician along with a nurse, a pharmacist and/or a physiotherapist.

### Study population

The study took place in one of the 16 French PAERPA region. The Valenciennois-Quercitain is a geographical area in the North of France with a surface area of 1040 km^2^ and a total of 31 520 people aged 75 years or older living in this area. When PAERPA was initiated, there were 348 family doctors, 148 dispensary pharmacies, 390 community nurses, five social networks (local information and coordination center) and several home help services for the elderly. All family physicians and community pharmacists and nurses in the Valenciennois-Quercitain were contacted by the PAERPA support center at the initial phase of PAERPA and during the project. HCPs who agreed and not agreed to participate in the device (PAERPA) were those who agreed or disagreed to perform at least one PDP or PFP for one of their older patient during the PAERPA pilot program.

The PAERPA support center provided us with the list of HCPs having agreed or refused to participate in the pilot program. These HCPs were eligible for our qualitative study. A selection of those HCPs was recruited by purposive sampling (based on age, sex, and urban/rural location) and then contacted by phone. HCPs who agreed to participate were included in our study. The recruitment process continued until no new information was generated in study interviews.

### Data collection

The data collection process is summarized in ***Figure 1***.

During the first phase of the study, we performed qualitative, semi-structured interviews with (i) hospital physicians (HP) involved in PDPs (interviewer: EN); (ii) family physicians (FP) involved in PDPs (interviewer: ML); (iii) community pharmacists (CPh) involved in PDPs (interviewer: AD); (iv) nurses (N) involved in PFPs (interviewers: PH and GA); (v) family physicians involved in PFPs (interviewer: CR); (vi) family physicians who refused to participate in the PAERPA pilot program (interviewer: FS).

Each of the interviewers drafted an interview guide, which (after approval by the steering committee) could be modified as the series of interviews progressed. The interviews took place at the HCP’s office or pharmacy. The interviews were audio-recorded, with the interviewees’ consent. The interviewer and the interviewee were alone together during the interviews (i.e. no observers were present). Data were collected until two successive interviews did not provide additional insights.

### Data analysis and coding

The data analysis process is summarized in ***Figure 1***.

Each interview was fully and anonymously transcribed and then reported in verbatim. For each qualitative study, the investigator in charge coded and analyzed the data using an anchored theorizing approach using Nvivo® software (QSR International Pty Ltd, Melbourne, Australia). The relevant categories and relationships within the verbatim were identified and then organized into “concept trees”, in order to develop a theory explaining the HCPs’ behaviors and feelings. All the verbatim data were checked by one of the other interviewers. Any issues during data analysis and coding were discussed within the steering committee and resolved by consensus.

### Synthesis

Two members of the steering committee (LA, CDM) compared the results of the six qualitative sub-studies and identified elements corresponding to a facilitator or obstacle to the implementation of integrated care. Each element identified in one sub-study was cross-referenced with the elements identified in the other sub-studies. If an element related to a barrier or facilitator was found in at least three sub-studies, then it was retained as an item shared by the HCPs. For each of these items identified as common facilitators and/or barriers, all associated verbatim were reviewed, analyzed and compared. This second comparative analysis focused on identifying the convergences and/or divergences between the different HCPs for a given concept. The two reviewers separately and independently determined how the concept was globally expressed by a given category of HCPs. They then cross-checked their results and validated their proposals with the interviewers who had conducted the interviews. Disagreements in the comparative analysis were resolved by discussion or consensus, and an opinion of the steering committee was sought where appropriate. The results of this second analysis were synthesized by the member of the steering committee (LA, CDM) and the interviewers validated the conclusions of this synthesis.

## Results

### Study population

**Figure 2:**
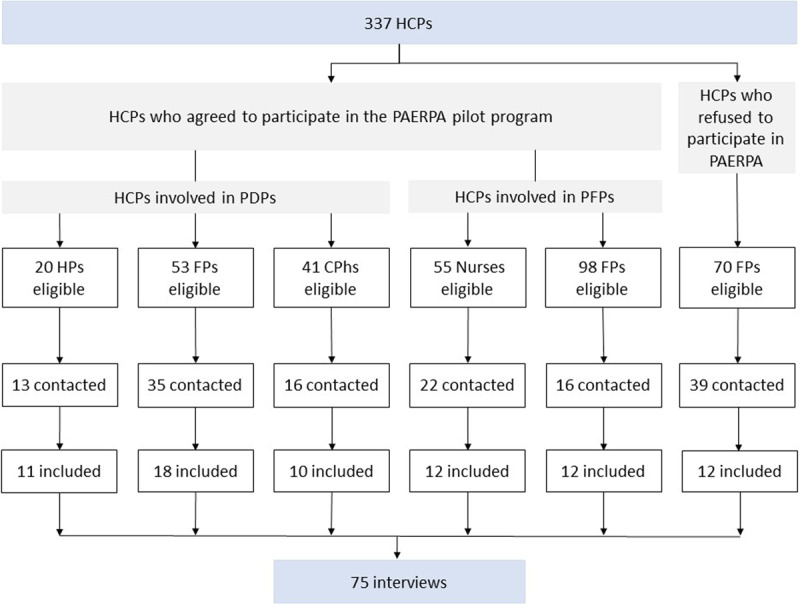
Study flow chart. HP = hospital physician; FP = family physician; CPh = community pharmacist; PDP = personalized drug plan; PFP = personalized frailty plan.

A total of 337 HCPs were eligible for inclusion in the qualitative surveys; 141 were contacted, and 75 agreed to be interviewed (phase 1, ***Figure 1***). Four different categories of HCP were interviewed: hospital physicians, family physicians, nurses and community pharmacists (indicated hereafter in the verbatim as HPs, FPs, Ns, and CPhs, respectively. The study flow chart is shown in ***Figure 2***.

The characteristics of the HCPs who participated in the present study are summarized in the Supplementary Data 2.

### Synthesis of the six qualitative surveys

The synthesis of the six qualitative surveys is presented in ***Table 1***.

**Table 1:** Results of the six qualitative surveys: common themes.


	HCPS WHO AGREED TO PARTICIPATE IN THE PAERPA PROGRAM					HCPS WHO REFUSED TO PARTICIPATE IN THE PAERPA PROGRAM

	HCPS INVOLVED IN A PDP			HCPS INVOLVED IN A PFP		PDPS AND PFPS

	HPS	FPS	CPHS	FPS	NURSES	FPS

Interest in geriatric care	Moderate	Moderate	High	High	Not mentioned	Absent

Frustrated by care for elderly patients	Moderate	Moderate	Not mentioned	High	High	Absent

Level of information about the project	Insufficient	Insufficient	Insufficient	Insufficient	Insufficient	Insufficient

Communication between HCPs	Little or no change	Little or no change	Little or no change	Little or no change	Little or no change	–

Feedback on patient outcomes	Insufficient	Insufficient	Insufficient	Insufficient	Insufficient	–

Role of the care coordinator	–	Essential	Essential Barrier to direct communication with HCPs	Essential	Essential Catalyst for communication between HCPs	–

Personal benefit	Time savings. Greater knowledge about pharmacology in older patients	Time savings. Task delegation	Enhancing the status of the health professions	Time savings. Task delegation.	Time savings. Task delegation. Enhancing the status of the health professions. Training	Extra workload and a waste of time. Intrusiveness. Judgment of personal practices

Benefit for the patient	High: medication review by an expert	High: medication review by an expert	High	Not often mentioned	High	Intrusion into the physician-patient relationship


HP = hospital physician; FP = family physician; CPh = community pharmacist; PDP = personalized drug plan; PFP = personalized frailty plan.

Barriers and facilitators shared by HCPs were grouped into four main topics: (i) awareness of and/or interest in geriatric medicine and in team working, (ii) the presence of a care coordinator; (iii) the provision of information about the program and about the patient, and communication between HCPs, and (iv) personal benefits for the HCPs and the patients. All four topics were mentioned explicitly by the HCPs who agreed to participate in PAERPA and also (with opposing views) by family physicians who refused to participate.

### Awareness and/or interest in geriatric medicine and collaborative work

The HCPs who had agreed to participate in the PAERPA pilot program felt concerned about geriatric medicine and/or were frustrated with the way that frail older patients had been managed before the start of the PAERPA program. Although there were several reasons for this frustration, a lack of time, a lack of resources and poor coordination were mostly frequently mentioned. The integrated care pathway proposed by the PAERPA pilot program enabled multidisciplinary, collaborative work and expertise-sharing. The HCPs were in favor of the PAERPA program because it addressed some of their problems.

“The problem with older patients with many concomitant conditions (…) is that they are difficult to manage” (FP7); “My view is that the more you look at people, the more things you see. The more people are involved, the better the system works (…) And then there will be people who have (…) skills that I don’t have. (…) I consider that these people will contribute in areas in which I am not competent… “(FP26); “It’s my main activity – it accounts for 90% of my patients” (N9).

Family physicians who refused to participate in the PAERPA pilot program were either (i) reluctant to collaborate within a network, (ii) not interested in or aware of geriatric medicine, or (iii) satisfied with their current practice. They were unwilling to change their current practice, and they perceived coordination as a way of spying on their activities.

“Don’t you care that much about geriatric patients, then? (interviewer). I had some elderly patients – so many that I became sick of them (FP37); “I didn’t see the point (…), you’re stating the obvious” (FP35); “I don’t need a government employee to come and tell me what to do” (FP32).

### The presence of a care coordinator

The presence of a care coordinator was always described by HCPs as being an essential success factor for the PAERPA pilot program. By dealing with administrative tasks, the care coordinator saved time for the HCPs, which was much appreciated.

“The care coordinator is essential. Otherwise the program would waver” (CPh9); “I found that the only useful thing in the PAERPA program was the care coordinator” (FP27); “Well, it would be more complicated without her [*the care coordinator*] because she manages everything. It would mean extra work for us” (N3).

The care coordinator’s involvement in communication between HCPs was appreciated by family physicians and nurse, although pharmacists appeared to prefer more direct communication.

“There’s not much dialogue with the physicians… She [*the care coordinator*] is the one who deals with them” (CPh8); “I reckon that if she wasn’t there to coordinate everything, we’d always be calling the physicians to update them and give them our opinion” (CPh3).

### The provision of information about the program and about the patient, and communication between HCPs

A lack of information and communication was the main weakness reported by all the HCPs interviewed. This lack was felt at three different levels.

Firstly, many HCPs considered that the initial presentation of the PAERPA pilot program was not sufficiently detailed. This problem appeared to have dissuaded some HCPs from participating in the pilot program.

“So you’d never heard about the PAERPA program – not even from other physicians? (interviewer) “No” (FP33); “Because it was very vague at first (…) Well, it wasn’t very well presented” (FP39).

Even HCPs who were satisfied with the initial presentation would have liked to have received reminders about the program’s characteristics, operation and resources.

“No “booster doses” since the first “shot”…” (HP4).

Secondly, a lack of information about the patient (e.g. the lack of a shared folder for patient assessments and follow-up) was reportedly a difficulty. Some HCPs complained about a lack of feedback on patient follow-up and the effectiveness of the interventions.

“I mean, I didn’t get any feedback either” (N8); “… that’s what is frustrating because we don’t get any feedback on the patients (…)” (HP4); “We never get feedback on what’s going on. We never get feedback from the physician, in fact, and so we don’t really know what the situation is” (CPh9).

Thirdly, the HCPs noted the absence of real changes in the nature and modalities of inter-HCP dialogue.

“What do you think of the communication between hospital physicians on one hand, and nurses, pharmacists and other hospital-based stakeholders on the other?” (interviewer) “I don’t have any contact with them – we have a lot of dialogue between pharmacists all the time but it’s not through the PAERPA program (FP3)”; “During the PAERPA program, I did not have much contact with the hospital physicians. Everyone does their bit on their own” (CPh6); “(*concerning inter-professional dialog*) Well, there’s no dialogue between HCPs – none” (N2); “But we already communicate, so it doesn’t change anything” (N4).

### Personal benefits for the HCPs and the patients

The HCPs considered that their involvement in the program was beneficial for their patients and increased the quality of the medical and psychosocial care provided.

“(…) A patient in the PAERPA program receives better care than a patient outside the program” (FP16); “We really studied the patient from the start of the process to the end” (N4); “This aspect of knowledge sharing and medical education is interesting and was much appreciated” (HP11).

All HCPs participating in the pilot program also noted personal benefits on several levels: time savings, a decrease in the administrative workload, improved practice, and a better image of their profession. In particular, they considered that the possibility of delegating administrative tasks to the care coordinator saved time and was essential. Almost all the HCPs (and especially the participating family physicians) mentioned that the presence of a coordinator was a facilitating factor.

“It saved me a lot of time” (HP1); “She [the care coordinator] stole my work – but luckily for me because otherwise it would have required a lot of energy!” (FP21); “PAERPA also enables me to delegate tasks” (FP24); “If someone else manages it for us, then why not?” (FP39); “[*The PAERPA project*] gives the patient another vision of the pharmacist; yes, people are starting to see that we are not just people sells boxes of pills but that we help people to stay healthy, etc.” (CPh10)

Conversely, family physicians who did not participate in the PAERPA pilot program identified administrative work as one of the main obstacles.

“The project appeared to be very time-consuming. That’s what scared me (…)” (FP40); “It looked like it would generated a huge amount of extra work for me – something that would have been impossible with my current workload (…)” (FP41).

## Discussion

The present results showed that the implementation of an integrated care pathway for frail older patients must take account of four important issues for HCPs. These results were based on interviews with hospital physicians, family physicians, nurses, and community pharmacists – including some who had refused to participate in the PAERPA program. The four topics related to barriers and facilitators shared by HCPs were: (i) awareness of and/or interest in geriatric medicine and in team working, (ii) the presence of a care coordinator, (iii) communication about the program, about patient health outcomes and between HCPs, and (iv) personal benefits of involvement in the program for HCPs and patients.

Previous studies have shown that family physicians who are invited to participate in an integrated care pathway program must be aware of the program’s theme (e.g. frailty or diabetes mellitus). In a study of 22 family physicians having participated in the System of Integrated Care for Older Persons project, Da Stampa et al. found that those with a large number of older patients and/or who were interested in an integrated care pathway were more committed [1,2,3]. This result was also observed in other qualitative studies of family physicians having participated in integrated care pathways for diabetes, cardiovascular disease, and palliative care [6,7,8]. Our present results confirm and extend these findings. Integrated care is not solely based on the level of commitment by family physicians; it also involves other HCPs. Our study showed that the other participating HCPs must also be interested in geriatric medicine and teamwork. Conversely, family physicians who had refused to participate in the PAERPA program were not interested in these topics and were reluctant to collaborate within a network. Our results indicate that not all HCPs are motivated by participation in an integrated care pathway. The target HCPs should probably have been screened before the initiation of this type of project [3,4,5,6,8,18]. Furthermore, our results showed that all type of HCP perceived individual benefits for themselves and for their patients. With a view to increasing levels of motivation, these benefits should probably be highlighted when the program is first presented to the HCPs.

The value of a care coordinator has been extensively studied in the field of chronic disease [19,20,21]. In the context of integrated care for older adults, Sheaff et al. have shown that both patients and caregivers were very satisfied with the care coordinator [22]. This result has been found in qualitative studies of family physicians and older patients [3,12,23]. The present study showed that other HCPs (pharmacists, nurses, and hospital physicians) also perceived the care coordinator as being a key success factor for integrated care pathways. All the participants highlighted the care coordinator’s value in reducing the administrative burden and generating time savings. In France, the care of frail older people is often compartmentalized, with fragmented healthcare pathways, often represented by a succession of assessments and procedures. A full coordination between the various HCPs or between hospital and community settings is lacking [24,25]. The French PAERPA experiment has sought to provide a response to the problems posed by this fragmentation of care. The strong need for coordination identified in our study may be related to this French context and the poor inter-professional communication between HCPs in France. This may explain why the presence of a care coordinator was appreciated by all HCPs.

Several studies have shown that high-quality communication and information provision is essential for the implementation and sustainability of integrated care pathways [3,7,8,12,26,27]. A review of eight studies of integrated care in the elderly highlighted communication (especially between HCPs) as a key success factor. Shared access to electronic health records is known to optimize information flows and communication, and should be a priority within integrated care pathways [8,12]. Our results confirmed the literature data, and added details. We identified three levels of communication that should be taken into account: information about the program, information about the patient, and inter-HCP communication. Our results also showed that program communication must target and integrate all the healthcare professions, and that the HCPs’ feelings about these three levels of communication may vary. The sole implementation of shared-access electronic health records may not be able to meet all these challenges; additional technical and organizational solutions are probably required at each of the three levels identified here.

Our results can be placed in the proposed conceptual framework for the analysis of barriers and facilitators in the implementation of integrated care, containing the three macro, meso, micro levels. Our results were mainly at the micro level, including aspects of shared values and understanding, engagement, and communication. Some meso-level aspects were found in the organization of the systems and in the presence of the care coordinator. This is related to the fact that our study focused on integrated care pathways in PAERPA, which covered only part of Kodner’s definition of integrated care. The PAERPA pilot project included other levels, such as adjustments at the macro level (modification of care regulation decrees) and the meso level (financing, organization, motivation for change). However, the objective of our study was to focus on the health professionals and not on the whole PAERPA process.

The present study had a number of strengths, including the diversity of the HCPs interviewed (family physicians, hospital physicians, nurses, and community pharmacists), the inclusion of family physicians who had refused to participate in the PAERPA program, COREQ-compliant reporting (30 out of 32 criteria; see Supplementary Data 1), the validation and coordination of the qualitative studies by a steering committee, the interviewers’ level of training, and the double proofreading of all interviews. To the best of our knowledge, this study is the largest qualitative study of the barriers to and facilitators of HCP involvement in an integrated care pathway for frail older patients [3,12,23,28,29,30].

The study also had some limitations. Firstly, we did not interview any patients or caregivers. Secondly, we did not interview nurses having refused to participate in the PAERPA program. Thirdly, the study was limited to a population of HCPs in a single region of France. Caution will therefore be required when extending the results to other HCPs or other study areas. Relative to France as a whole, the Hauts-de-France region is characterized by a young population, high levels of social deprivation, and high morbidity and mortality rates. Thirdly, in PAERPA, the realization of PDPs and PFPs had to be centered on the family physician. Consequently, our study is based on a GP-centered model and did not include other services available in PAERPA. Our conclusions should be cautiously extended to other models of integrated care. Lastly, an Internet-based platform for dialogue and information exchange between HCPs in the Hauts-de-France region was initially planned as part of the PAERPA program but could not be implemented. The importance of communication may therefore have been over-emphasized by the HCPs because of this problem.

## Conclusion

We identified four categories of barriers or facilitators influencing the readiness of HCPs to implement integrated care pathways for older patients, regardless of whether or not the HCPs had agreed to participate in PAERPA program: (i) awareness of and/or interest in geriatric medicine and team working, (ii) the presence of a care coordinator, (iii) the provision of information about the project, feedback on the patient outcomes, and communication between HCPs, and (iv) personal benefits for the HCP and the patient. The issues related to these four barriers and facilitators should be taken into account when designing integrated care projects for older people.

## Additional Files

The additional files for this article can be found as follows:

10.5334/ijic.5483.s1 Supplementary data 1. Consolidated criteria for reporting qualitative studies (COREQ): 32-item checklist.

10.5334/ijic.5483.s2 Supplementary data 2. Characteristics (age and sex) of the HCPs (item 16 of the COREQ checklist: domain 2, Settings, Sample description).
