# Supplementary material for: Barriers and Facilitators in the Uptake of Integrated Care Pathways for Older Patients by Healthcare Professionals: A Qualitative Analysis of the French National “Health Pathway of Seniors for Preserved Autonomy” Pilot Program: Barriers and Facilitators for Adhesion of Healthcare Professionals in Integrated Care for Older Patients: A Qualitative Assessment Based on the French National Experiment: ‘Health Pathway of Seniors for Preserved Autonomy’ (PAERPA)
Source: Int J Integr Care. 2021 Apr 22;21(2):7. doi: 10.5334/ijic.5483 (PMC8064286; doi:10.5334/ijic.5483)
Supplement: Supplementary data 2. — Characteristics (age and sex) of the HCPs (item 16 of the COREQ checklist: domain 2, Settings, Sample description). [file ijic-21-2-5483-s2.pdf]

**Supplementary data 2**

Characteristics (age and sex) of the HCPs (item 16 of the COREQ checklist: domain 2, Settings, Sample description).

| Healthcare professional | Age | Sex |
|---|---|---|
| Family physician 1 | 50 | M |
| Family physician 2 | 63 | M |
| Family physician 3 | 61 | M |
| Family physician 4 | 52 | M |
| Family physician 5 | 43 | F |
| Family physician 6 | 49 | M |
| Family physician 7 | 43 | F |
| Family physician 8 | 57 | M |
| Family physician 9 | 30 | M |
| Family physician 10 | 59 | M |
| Family physician 11 | 58 | F |
| Family physician 12 | 62 | M |
| Family physician 13 | 45 | F |
| Family physician 14 | 60 | M |
| Family physician 15 | 60 | M |
| Family physician 16 | 42 | M |
| Family physician 17 | 61 | M |
| Family physician 18 | 65 | M |
| Family physician 19 | 63 | M |
| Family physician 20 | 68 | M |
| Family physician 21 | 51 | F |
| Family physician 22 | 52 | M |
| Family physician 23 | 43 | M |
| Family physician 24 | 37 | M |
| Family physician 25 | 49 | M |
| Family physician 26 | 37 | M |

| | | |
|---|---|---|
| Family physician 27 | 47 | M |
| Family physician 28 | 41 | F |
| Family physician 29 | 61 | F |
| Family physician 30 | 33 | M |
| Family physician 31 | 40 | M |
| Family physician 32 | 63 | M |
| Family physician 33 | 64 | M |
| Family physician 34 | 60 | M |
| Family physician 35 | 55 | M |
| Family physician 36 | 54 | F |
| Family physician 37 | 58 | M |
| Family physician 38 | 54 | M |
| Family physician 39 | 43 | M |
| Family physician 40 | 46 | M |
| Family physician 41 | 49 | M |
| Family physician 42 | 66 | M |
| Hospital practitioner 1 | 40 | M |
| Hospital practitioner 2 | 53 | F |
| Hospital practitioner 3 | 36 | F |
| Hospital practitioner 4 | 45 | F |
| Hospital practitioner 5 | 56 | F |
| Hospital practitioner 6 | 54 | F |
| Hospital practitioner 7 | 42 | M |
| Hospital practitioner 8 | 55 | M |
| Hospital practitioner 9 | 58 | M |
| Hospital practitioner 10 | 54 | F |
| Hospital practitioner 11 | 59 | M |
| Community pharmacist 1 | 55 | F |
| Community pharmacist 2 | 33 | F |
| Community pharmacist 3 | 51 | F |
| Community pharmacist 4 | 55 | M |

| | | |
|---|---|---|
| Community pharmacist 5 | 30 | F |
| Community pharmacist 6 | 48 | F |
| Community pharmacist 7 | 44 | M |
| Community pharmacist 8 | 59 | M |
| Community pharmacist 9 | 41 | F |
| Community pharmacist 10 | 37 | M |
| Nurse 1 | 54 | F |
| Nurse 2 | 69 | F |
| Nurse 3 | 65 | F |
| Nurse 4 | 57 | F |
| Nurse 5 | 34 | F |
| Nurse 6 | 30 | F |
| Nurse 7 | 54 | F |
| Nurse 8 | 43 | F |
| Nurse 9 | 51 | F |
| Nurse 10 | 36 | F |
| Nurse 11 | 42 | M |
| Nurse 12 | 52 | F |

**Interview duration: Item 21 of the COREQ checklist: domain 2, data collection, duration.**

| Healthcare professional | Interview duration (minutes) |
|---|---|
| Family physician 1 | 23 |
| Family physician 2 | 19 |
| Family physician 3 | 13 |
| Family physician 4 | 21 |
| Family physician 5 | 40 |
| Family physician 6 | 21 |
| Family physician 7 | 23 |
| Family physician 8 | 30 |
| Family physician 9 | 19 |
| Family physician 10 | 22 |
| Family physician 11 | 19 |
| Family physician 12 | 33 |
| Family physician 13 | 8 |
| Family physician 14 | 25 |
| Family physician 15 | 24 |
| Family physician 16 | 17 |
| Family physician 17 | 14 |
| Family physician 18 | 7 |
| Family physician 19 | 6 |
| Family physician 20 | 9 |
| Family physician 21 | 8 |
| Family physician 22 | 10 |
| Family physician 23 | 20 |
| Family physician 24 | 15 |
| Family physician 25 | 13 |
| Family physician 26 | 16 |
| Family physician 27 | 18 |
| Family physician 28 | 10 |

| | |
|---|---|
| Family physician 29 | 15 |
| Family physician 30 | 10 |
| Family physician 31 | 28 |
| Family physician 32 | 13 |
| Family physician 33 | 6 |
| Family physician 34 | 18 |
| Family physician 35 | 19 |
| Family physician 36 | 8 |
| Family physician 37 | 26 |
| Family physician 38 | 11 |
| Family physician 39 | 25 |
| Family physician 40 | 11 |
| Family physician 41 | 22 |
| Family physician 42 | 13 |
| Hospital practitioner 1 | 18 |
| Hospital practitioner 2 | 14 |
| Hospital practitioner 3 | 15 |
| Hospital practitioner 4 | 13 |
| Hospital practitioner 5 | 14 |
| Hospital practitioner 6 | 18 |
| Hospital practitioner 7 | 16 |
| Hospital practitioner 8 | 16 |
| Hospital practitioner 9 | 13 |
| Hospital practitioner 10 | 17 |
| Hospital practitioner 11 | 22 |
| Community pharmacist 1 | 45 |
| Community pharmacist 2 | 31 |
| Community pharmacist 3 | 58 |
| Community pharmacist 4 | 60 |
| Community pharmacist 5 | 37 |
| Community pharmacist 6 | 32 |

| | |
|---|---|
| Community pharmacist 7 | 28 |
| Community pharmacist 8 | 26 |
| Community pharmacist 9 | 27 |
| Community pharmacist 10 | 43 |
| Nurse 1 | 35 |
| Nurse 2 | 38 |
| Nurse 3 | 50 |
| Nurse 4 | 42 |
| Nurse 5 | 44 |
| Nurse 6 | 40 |
| Nurse 7 | 43 |
| Nurse 8 | 40 |
| Nurse 9 | 40 |
| Nurse 10 | 29 |
| Nurse 11 | 36 |
| Nurse 12 | 42 |
